# The Influence of Physical Activity on the Bioactive Lipids Metabolism in Obesity-Induced Muscle Insulin Resistance

**DOI:** 10.3390/biom10121665

**Published:** 2020-12-12

**Authors:** Monika Imierska, Adam Kurianiuk, Agnieszka Błachnio-Zabielska

**Affiliations:** Department of Hygiene, Epidemiology and Metabolic Disorders, Medical University of Bialystok, 15-089 Bialystok, Poland; m.imierska@gmail.com (M.I.); adam.kurianiuk@umb.edu.pl (A.K.)

**Keywords:** physical activity, lipid metabolism, insulin resistance, type 2 diabetes, skeletal muscle

## Abstract

High-fat diet consumption and lack of physical activity are important risk factors for metabolic disorders such as insulin resistance and cardiovascular diseases. Insulin resistance is a state of a weakened response of tissues such as skeletal muscle, adipose tissue, and liver to insulin, which causes an increase in blood glucose levels. This condition is the result of inhibition of the intracellular insulin signaling pathway. Skeletal muscle is an important insulin-sensitive tissue that accounts for about 80% of insulin-dependent glucose uptake. Although the exact mechanism by which insulin resistance is induced has not been thoroughly understood, it is known that insulin resistance is most commonly associated with obesity. Therefore, it is believed that lipids may play an important role in inducing insulin resistance. Among lipids, researchers’ attention is mainly focused on biologically active lipids: diacylglycerols (DAG) and ceramides. These lipids are able to regulate the activity of intracellular enzymes, including those involved in insulin signaling. Available data indicate that physical activity affects lipid metabolism and has a positive effect on insulin sensitivity in skeletal muscles. In this review, we have presented the current state of knowledge about the impact of physical activity on insulin resistance and metabolism of biologically active lipids.

## 1. Introduction

Obesity is commonly defined as a state of abnormal or excessive fat accumulation leading to health deterioration [1]. It is a major risk factor in the development of many non-communicable diseases, i.e., cardiovascular diseases (CVD), hypertension, stroke, coronary heart disease, gallbladder diseases, and cancer. It is also associated with a higher probability of disability or premature death due to type 2 diabetes (T2D) [2]. The epidemics of obesity has become a great challenge for modern medicine. This problem affects all age groups in both, developed and developing countries [2]. Over the past 30 years, the number of obese people in many countries has quadrupled. If current trends continue, it is estimated that by 2030 38% of the world’s adult population will be overweight and another 20% will be obese [3]. The relationship between obesity and T2D has been known for decades. It is mainly based on the ability of obesity to induce insulin resistance (IRes), which is the most important etiological feature of T2D and is also associated with a wide range of other pathophysiological consequences [4]. T2D is a disease that nowadays affects 463 million people around the world. It is projected, that in 2040, there will be more than 700 million people suffering from diabetes worldwide [5]. The reasons for these forecasts are primarily the increasing number of obese people and the longer life expectancy of the population [6]. T2D is often associated with other complications, such as cardiovascular diseases, diabetic neuropathy, nephropathy, and retinopathy. These disorders decrease the quality of life and cause severe economic and social burdens [7]. Large percentage of obese children is also alarming. It could cause significant problems for healthcare systems in the oncoming decades [8]. Type 2 diabetes is a civilization disease associated with functional disorders in all main organs that are involved in glucose and lipids metabolism, including skeletal muscle, adipose tissue, liver, and pancreatic β–cells [9]. The basis of T2D is insulin resistance described as a weakened response of peripheral tissue to insulin, which in turn leads to impaired glucose homeostasis in the body [10]. The link between insulin resistance and T2D has been intensively studied for over half a century. IRes is considered to be the most powerful predictor of future development of type 2 diabetes mellitus [11].

Recent data suggest that physical activity may have especially favorable effects among patients with insulin resistance, metabolic syndrome, T2D or obesity [12]. Regular exercise provides many benefits like improvements in blood glucose control and ability to prevent or delay type 2 diabetes. Physical activity improves lipid metabolism and blood pressure, it may also reduce total daily insulin requirements in people on insulin treatment and is at least as effective in diabetes prophylaxis as medicines [13].

## 2. Insulin Signal Transduction Pathway and Mechanisms of Insulin Resistance

Adipose tissue has the ability to expand by a combination of enlarging existing adipocytes (hypertrophy) and increasing their number (hyperplasia) [14]. In the state of “normal” energy excess, adipose tissue expansion is achieved by formation and differentiating of adipose precursor cells instead of redirecting fat into mature adipocytes. Nonetheless, in obesity, this process could become greatly deteriorated. Permanent calorie overload leads to changes in the preadipocytes commitment and adipose tissue adipogenesis [15]. Redirecting fat to mature adipocytes causes the storage capacity of adipose tissue to reach its maximum level. It then leads to increased plasma lipids [16]. Chronically elevated plasma free fatty acid (FFA) concentration causes their accumulation in organs not intended for fat storage e.g., in the muscles. This phenomenon is called ectopic fat accumulation and may lead to systemic insulin resistance and type 2 diabetes [17,18,19]. Skeletal muscle is the largest tissue in the human organism and plays a pivotal role in locomotion and entire body metabolism. It has been identified as the primary tissue in glucose metabolism, accounting for 80% of insulin-stimulated glucose uptake [20,21]. Skeletal muscle insulin resistance, an important feature of type 2 diabetes, is caused by a reduced ability of muscle to respond to circulating insulin and has been related with intracellular lipid accumulation [22,23]. Randle et al. [24] in 1963 suggested the existence of a glucose-fatty acid cycle in which glucose and lipids are competitive substrates for oxidation in muscle and the availability of FFA results in glucose metabolism setback. However, lipid infusion studies have shown a two to four-hour delay between the increase in plasma FFA concentration and the onset of insulin resistance suggesting that elevated FFA level is not directly responsible for insulin resistance [25,26,27]. Currently, it is suggested that in the state of obesity, the concentration of fatty acids in the plasma increases and, thus, increases their supply to skeletal muscles. This, in turn, leads to an elevation in the synthesis of intracellular lipids, including biologically active lipids: long-chain acyl-CoA, ceramides and diacylglycerols. The accumulation of these lipids leads to inhibition of the insulin pathway, which results in a decrease in muscle glucose uptake and, consequently, an increase in blood glucose concentration [28].

### 2.1. Insulin Signaling Pathway

**Figure 1:**
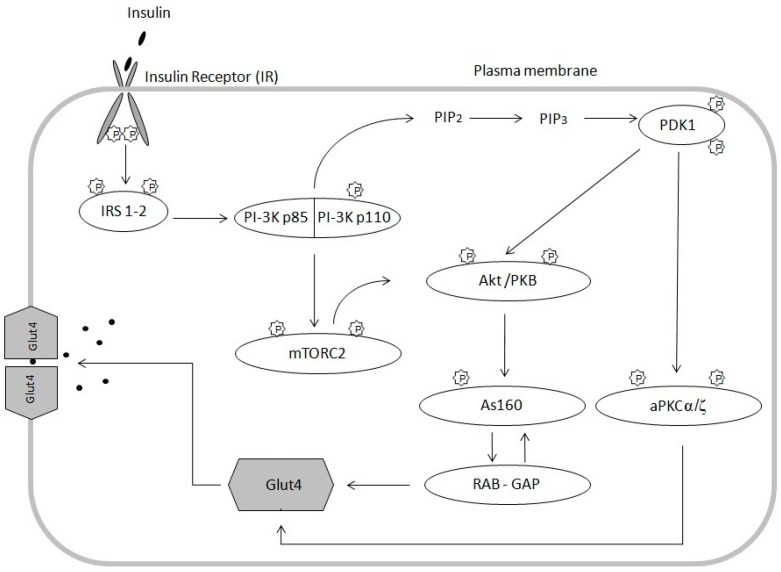
Insulin signaling pathway; Insulin stimulates the insulin receptor (IR), leading to phosphorylation of insulin receptor substrate (IRS). Recruitment of phosphatidylinositol 3-kinase (PI3K), generated of the second messenger PIP3, leading to the recruitment of Akt and subsequent phosphorylation by the upstream mediators PDK1 and mTORC2. PDK1 phosphorylates and activates PKC-ζ and α isoforms. Activated Akt phosphorylates Akt substrate 160 (AS160), thereby inactivating its Rab-GAP domain and preventing the hydrolysis of GTP to GDP by Rab proteins. This results in processes promoting the translocation of GLUT4 to the cell surface.

The pivotal action of insulin is to stimulate glucose uptake into cells by increasing the translocation of the glucose transporter 4 (GLUT4), from intracellular storage to the plasma membrane [29]. Insulin controls glucose uptake by acting on tyrosine kinase of insulin receptor. It is composed of two extracellular α subunits and two transmembrane β subunits combined together by disulfide bonds. Binding of insulin to the α subunit induces a conformational change resulting in the autophosphorylation of tyrosine residues present in the β subunit (Figure 1). Autophosphorylation of all tyrosine residues in the β subunit is needed for the full activation of the insulin receptor [30]. Receptor activation leads to phosphorylation of key tyrosine residues on signaling molecules, including Shc, Grb2 and insulin receptor substrates (IRS). IRS are scaffolding proteins and the two most predominant IRS involved in metabolic regulation in human skeletal muscles are IRS-1 and IRS-2 [31]. IRS 1-2 are phosphorylated on multiple tyrosine residues that form binding sites for intracellular molecules containing Src-homology 2 (SH2) domains, such as the p85 regulatory subunit of PI3 kinase (PI3-K), which is bound to the p110 catalytic subunit as a heterodimer [32]. The activity of PI3 kinase leads to the production of phosphatidylinositol bisphosphate (PIP2) and phosphatidylinositol triphosphate (PIP3). The PIP2 and PIP3 generation is necessary for full protein kinase B activation. Protein kinase B (PKB), also known as Akt (Akt/PKB), is phosphorylated at Thr308 in the catalytic domain by phosphinositide-dependent kinase-1 (PDK1) that interacts with the second messengers products PIP2 and PIP3. PDK1 directly phosphorylates and activates protein kinases C (PKC). PKC-ζ and α isoforms (PKC-ζ/α) are other signaling proteins involved in this process [33].

Complete activation of protein kinase B requires more phosphorylation steps in response to insulin beyond PDK-1 activity. DNA-dependent protein kinase (DNA-PK) [34] or mTOR: Rictor: GbL complex [5] phosphorylates of Akt at Ser473 [31,35,36,37]. Next, in response to insulin, Akt substrate, protein of 160 kDa (AS160), is phosphorylated. AS160 contains Rab GTPase activating protein (GAP) domain that is phosphorylated at several serine and threonine residues. This mechanism is dependent on phosphorylation of AS160 in response to insulin that leads to either inactivation of the Rab GAP function or redistribution of AS160 from the LDM compartment. In this way, Rab proteins are involved in GLUT4 vesicle translocation [31,38]. The presented model of insulin signaling indicates that insulin signaling pathway includes many signaling proteins that coordinate the translocation of GLUT4 to the cell membrane, which facilitates glucose transport to myocytes [39]. Inhibiting one of the stages in insulin signaling pathway, prevents GLUT4 from being translocated to the cell membrane. As a result, insulin-dependent cellular glucose uptake decreases, thereby inducing insulin resistance [40]. The widely accepted theory says, that the major cause for inducing muscle insulin resistance is fatty acid oxidation disorder. As a result, long-chain acyl-CoA (LCACoA) are redirected to the endoplasmic reticulum and cytosol, where they are used in the synthesis of biologically active lipids such as ceramides and diacylglycerols [41,42].

### 2.2. Obesity as a Factor of IRes

A traditional role attributed to adipose tissue is primarily the storage of energy, which is released when the body has too little of it. It should be stressed that this tissue also has a very complex metabolic function. It is essential for proper glucose homeostasis and it plays an important role in inflammatory processes [43]. Adipose tissue has a high energy storage capacity due to its ability to increase the amount of lipids stored in each cell (increase of lipogenesis over lipolysis) and by increasing the number of fat cells (processes of replication and preadipocyte differentiation) [44]. The ability to store excess energy has an obvious but, unfortunately, a short-term advantage for survival only in situations of insufficient food availability. Excess fat, such as in obesity, adversely affects long-term survival, which is associated with increased endocrine dysfunction of adipose tissue and metabolic disorders [45,46]. It is widely known that obesity is associated with insulin resistance and it is also a major risk factor for the development of T2D and cardiovascular diseases [47]. Adipocytes play a dual role in the development of insulin resistance. Firstly, adipocytes store fatty acids in the form of triacylglycerols, which can be released into the bloodstream and uptaken by other peripheral tissues where they can be a substrate in the de novo synthesis of biologically active lipids inhibiting the activity of the insulin pathway. Secondly, adipose tissue cells synthesize hormones that affect insulin sensitivity of other peripheral tissues [42]. 

When the balance between energy intake and expenditure is not maintained, or when there is a problem with excessive energy storage in the form of triacylglycerols in adipocytes, as it occurs in obesity, the plasma FFA concentration increase, as a result of their increased release from adipose tissue [48]. If the fatty acids are not oxidized, they will accumulate as triglycerides in other tissues and promote cellular lipotoxicity as well as mitochondrial dysfunction [49]. The phenomenon of lipid accumulation in organs not intended for fat storage, e.g., in the liver or muscles, is called ectopic fat accumulation [50]. A great importance of FFA in metabolic disorders results from the fact that it promotes IRes development by interacting with the insulin signal pathway [51,52]. On the other hand, adipose tissue IRes is manifested by a weakened effect of insulin on the inhibition of hormone-sensitive lipase (HSL) activity, which is responsible for the release of FFA from triacylglycerols stored in adipocytes [53], as a result of which the release of fatty acids increases, which in turn leads to the lipotoxicity [54]. In the fasting state, HSL is stimulated by hormones such as catecholamines that activate this enzyme through cAMP-dependent phosphorylation [55], while postprandial activity of this lipase is inhibited by insulin [56]. Therefore, HSL and insulin play an important role in the regulation of various metabolic disorders including lipotoxicity, T2DM, IRes, inflammations, steatosis, NAFLD, and obesity [57]. 

Despite the major role of HSL in adipocyte lipolysis, evidences show that HSL is not the only hormone-responsive lipase in adipocytes [58]. Studies reported about enzyme designated as adipose triglyceride lipase (ATGL) that take part in triglyceride-specific hydrolysis and therefore may play a relevant role in lipid metabolism as HSL. ATGL contributes to net adipocyte lipolysis and adipose tissue expression of ATGL is reciprocally regulated by nutritional status and by insulin in vitro and in vivo. It has been also demonstrated that adipose tissue expression of ATGL is evidently higher in insulin deficiency conditions [59]. As already mentioned, adipose tissue also has an important endocrine function It produces and secretes biologically active compounds—adipocytokine, including leptin, adiponectin, resistin, interleukin 6 (IL6), and tumor necrosis factor α (TNF α). In obesity, the production of many of these products is impaired and all these molecules have the ability to influence glucose metabolism [60,61]. Leptin plays a pivotal role in food and energy balance regulation. This protein, secreted mainly by white adipose tissue, can affect the central nervous system by reducing appetite and increasing metabolism of accumulated reserves. The concentration of circulating leptin is individually proportional to the amount of fat [62,63]. At first, scientists thought leptin would be the key to control obesity. Unfortunately, recent data have shown, that most cases of obesity are characterized by excessive leptin production and leptin resistance. This results in uncontrolled food intake and fat storage [64]. Important knowledge on the role of leptin in the regulation of energy balance was obtained during the studies conducted on ob/ob mice with mutations in the gene responsible for the production of this molecule. These experiments showed that obese mice (ob/ob) lacked a factor that could regulate obesity by modulating appetite and metabolism. It was also shown that the administration of leptin to ob/ob mice reversed their obesity phenotype and led to the conclusion that the physiological role of leptin is to reduce body weight and food intake [65,66]. Moreover, it has been shown that in metabolic diseases, such as obesity and diabetes, leptin signaling may be impaired, indicating its important role in the etiology and pathophysiological manifestations of these conditions [67]. However, leptin turned out to be not only a major regulator of energy balance, but also a hormone that modulates glucose homeostasis, neuroendocrine axes, the autonomic nervous system, memory, neuronal plasticity, and other biological functions. 

Adiponectin is the most common peptide hormone secreted by adipocytes. There is growing evidence that this protein is of great importance in regulating glucose and lipid homeostasis. Due to its ability to reduce triglyceride (TG) synthesis in skeletal muscles, activation of β-oxidation and increase of insulin efficiency in the liver, adiponectin has been classified as “antidiabetic” protein [68,69,70]. In obesity, a significant decrease in plasma adiponectin concentration is observed. The reduction of this protein plays a key role in obesity-related diseases, including insulin resistance/type 2 diabetes and cardiovascular diseases. This has been proven in studies where increased insulin sensitivity was observed following administration of adiponectin to rodents [71] and humans [72]. In addition, administration of adiponectin has also been shown to have anti-atherosclerotic and anti-inflammatory effects and, in some situations, to cause weight loss [73]. 

Another signal particle secreted especially by mature adipocytes and affecting the induction of insulin resistance is resistin. In diet-induced obesity, as well as in animal models of obesity and insulin resistance, the level of resistin significantly increases [74]. Experimental studies have demonstrated that resistin administration to mice impairs glucose uptake without reducing insulin secretion. It has also been shown that neutralization of this protein reduces hyperglycemia in obese and insulin-resistant mice by increasing insulin sensitivity. These data suggest that resistin is a unique hormone that has an insulin-antagonistic effect on glucose metabolism. It is suspected that resistin modulate one or more stages of the insulin signal pathway in insulin-responsive tissues [75]. The studies conducted so far show that resistin inhibits insulin-stimulated phosphorylation of protein kinase B (Akt protein) and glycogen synthase-3 kinase (GSK3) [76]. 

Other compounds that may contribute to the reduction of peripheral insulin sensitivity are proinflammatory cytokines. TNFα and IL-6 play an essential role in both the immune response and energy metabolism. In obesity, accompanied by chronic inflammation, these compounds are constantly secreted, which may affect the energy balance of the whole organism [77]. There is ample evidence that TNF-α is involved in the induction of obesity-related insulin resistance [78]. The influence of TNF-α on the development of insulin resistance has been confirmed both, in cell culture studies and animal studies. Studies on adipocyte and myocyte cultures have shown a decrease in insulin sensitivity under the influence of TNF-α. It is a result of the inhibition of phosphorylation insulin receptor (IR) and its substrates (IRS) [79,80,81] by TNF-α and lower expression of GLUT4, IRS-1, and IR [82,83,84]. In addition, increased expression of TNF-α has been observed in adipose tissue in various obesity models in rodents [85]. Neutralization of TNF-α in obese and insulin-resistant rats improves insulin receptor signaling increasing peripheral tissue sensitivity to insulin [80]. Studies on animals devoid of the TNF-α encoding gene with diet-induced obesity have shown significant improvement in insulin sensitivity. Moreover, mice deficient in TNF-α have lower levels of circulating FFA, which are a potential cause of the development of insulin resistance. The improvement of insulin-dependent glucose uptake in such conditions indicates that TNF-α is an important mediator of insulin resistance through negative influence of this cytokine on important sites of the insulin signaling pathway [86,87]. Interleukin 6 is another cytokine that appears to be involved in inducing insulin resistance. It is secreted mainly by cells of the immune system, adipose tissue and skeletal muscles. Many studies indicate that an increase in plasma IL-6 concentration is associated with obesity and type 2 diabetes. It has been shown that visceral adipose tissue in severely obese nondiabetic patients releases two to three times more IL-6 than subcutaneous adipose tissue [88]. Moreover, studies conducted on overweight women have shown a direct positive correlation between insulin resistance and circulating IL-6 levels [89]. It has also been shown that this cytokine increases hepatic triglyceride secretion and reduces lipoprotein lipase (LPL) activity in adipose tissue, which consequently enhances lipolysis and increases the level of circulating free fatty acids [90].

### 2.3. Role of Ceramides in IRes

Ceramides belong to a lipid group that consist of a sphingosine base that is linked to a fatty acid moiety. They create structural elements of the membrane lipid bilayer and act as substantial signaling molecules that are implicated in cellular processes [91,92]. Although they play important roles in cell biology, they are also thought to be involved in the development of insulin resistance [93,94]. Excessive FA influx into skeletal muscle may lead to exceeding the oxidative capacity of mitochondria which may result in increased production of biologically active lipids: ceramides and DAG [95,96]. Experimental studies revealed that ceramides are able to interfere with the insulin signaling pathway through maintaining protein kinase B in an inactive dephosphorylated state [97]. A consequence of ceramide action is the reduction of GLUT4 translocation to plasma membrane and a decrease in insulin-stimulated glucose uptake. Studies performed over the past decade have demonstrated that ceramide affects Akt/PKB activity [98,99,100]. The conducted studies indicate a large role of protein phosphatase 2A (PPA2) in mediating the inhibitory effect of ceramide on insulin-stimulated PKB activity. Ceramide-mediated inhibition of Akt/PKB consists of dephosphorylation of PKB by a serine/threonine phosphatase PP2A [101]. The other way that ceramide is known to promote insulin resistance is to reduce Akt/PKB phosphorylation through a process dependent on the activation of atypical protein kinase C (aPKC) λ/ζ isoforms [102,103]. Atypical protein kinase C serves as a molecular switch to activate the response associated with GLUT4 translocation/glucose transport during the actions of insulin in skeletal muscles and adipocytes. Researchers observed that the activation of aPKCs by insulin in skeletal muscles is defective in type 2 diabetic and insulin resistant humans, monkeys and rodents. This defect in aPKC activation seems to contribute to the diminution in insulin-stimulated glucose uptake [104]. It was concluded that the interaction between PKC λ/ζ and Akt/PKB requires the Akt/PKB pleckstrin homology domain (PH-domain) and that ceramide-activated PKC λ/ζ phosphorylates a threonine or serine residue at site 34 (dependent on Akt/PKB isoform) within this region [105]. As a result, diminished affinity of the PH-domain towards PIP-3 averts the recruitment of Akt/PKB to the plasma membrane and its subsequent activation. Based on these observations it has been suggested that the increase in intracellular ceramide content leading to activation of aPKCλ/ζ attenuates the recruitment of Akt/PKB to the plasma membrane as a result of interrupted PIP3 binding [105,106,107]. Consequently, insulin-dependent glucose uptake in muscle decreases in connection with inhibition of insulin pathway and weakened glucose transporter GLUT4 translocation. Ceramides have also been demonstrated to activate extracellular signal-regulated kinase 2 (ERK2), p38, JNK, IκB kinases (IκKβ) [108,109,110,111] and all of these enzymes have been implicated in the serine/threonine phosphorylation and inactivation of IRS-1 [112].

### 2.4. Role of Diacylglycerols in IR

Another biologically active lipid, the excess of which may be involved in the development of muscle insulin resistance, is diacylglycerol (DAG). The accumulation of DAG in skeletal muscles is part of the lipotoxicity hypothesis in the genesis of IRes [113,114]. Increased plasma FA concentration leads to the accumulation of intramyocellular acyl-CoAs and DAG, which activates protein kinases C (PKC) [115]. The activity of some isoforms of this enzyme interferes with the insulin pathway. It has been demonstrated that DAG accumulation is associated with the activation of protein kinase C-theta (PKC-θ) that is responsible for phosphorylation of Ser^307^ residue in insulin receptor substrates 1, what disrupts insulin signaling pathway [28,116]. It has been also found that DAGs containing C16:0, C18:0, C18:1, C18:2 or C20:4 fatty acids, are significantly associated with PKC- θ activation in obese and T2D individuals. [117]. DAG accumulation has resulted in a 70% decrease in the inhibitor of NFkβ (Ikβ-α) and Jun N-terminal kinase, that phosphorylates insulin-receptor substrates on serine residues. This observation is also relevant to the link between IRes and inflammatory status [26,118].

## 3. Effect of Exercise on Lipid Metabolism and Insulin Sensitivity

Nowadays there are various methods available for the treatment of T2D, both oral and insulin therapy. The best way to prevent obesity and related metabolic disorders is to change a lifestyle. In addition to a healthy diet, physical activity seems to play a key role. [6]. It is well known that regular exercise improves blood glucose control, which can prevent or delay type 2 diabetes. It also has a positive effect on lipid metabolism, blood pressure, cardiovascular events, and quality of life [13]. Physical activity that improves glucose homeostasis can be divided into aerobic exercise and anaerobic exercise (strength/resistance training). Aerobic training is defined as an activity in which many groups of muscles participate and is performed continuously and rhythmically. As the name suggests, this type of training is completely dependent on oxygen availability. In this type of exercise, the muscles need oxygen to generate energy in the form of adenosine triphosphate (ATP) from carbohydrates, fatty acids and amino acids [119,120]. Aerobic exercise enhances insulin sensitivity by increasing the translocation of glucose transporters to the plasma membrane, improving pathophysiological pathways associated with insulin resistance (reduction of adipokines in response to inflammatory and oxidative stress), and improving insulin signal transduction through various molecular pathways [121]. 

Contrary to aerobic exercise, short-term bodybuilding exercise (strength/resistance training) is anaerobic exercise [121,122]. The mechanisms underlying the positive effects of resistance training on glucose homeostasis and insulin sensitivity are similar to those in aerobic training. High intensity resistance training is effective in the treatment of diabetes in the population of elderly people at high risk of type 2 diabetes. This type of training significantly improves glycemic control, increases muscle mass while reducing abdominal obesity. Similar to aerobic exercise, anaerobic exercises have been shown to have a positive influence on the parameters associated with metabolic syndrome, such as glucose intolerance, hyperinsulinemia, hypertension, and hypertriglyceridemia [123]. Both types of exercise—resistance and aerobic—affect the increase of the rate of insulin-stimulated glucose uptake. It is worth noting that the improvement of glucose transport after resistance training is independent of a considerable increase in muscle mass. This means that this training does not have to cause an increase in skeletal muscle mass to improve glucose metabolism [124]. Another type of training that seem to be recognized as good way for countering insulin resistance is high-intensity interval training (HIIT). This type of training is conducted at maximum intensity in sessions of brief duration [125]. HIIT is able to serve as an effective alternate to traditional endurance training. This training induces similar changes in a scope of physiological, performance and health-related markers in both healthy and diseased populations [126]. HIIT improves blood pressure, cardiac function [127], oxidative stress, inflammation markers, and lipid metabolism [128]. Recent study shows, that HIIT seems to also be effective in improving insulin sensitivity, especially in those at risk of, or with, T2D [129]. However, the information towards its efficiency in insulin sensitization is inconsistent and molecular mechanism associated with the effect of HIIT on skeletal muscle of individuals with IRes has not been thoroughly investigated [130]. Presumably, the impact of HIIT on IRes is caused by higher skeletal muscle mitochondrial content and function [131]. It has been shown that HIIT is a potent activator of upstream signals to mitochondrial biogenesis such as PGC1-α (peroxisome proliferator-activated receptor gamma, coactivator 1 alpha) and TFAM (mitochondrial transcription factor A) [132,133].

### 3.1. Effect of Physical Activity on Insulin Signaling Pathway

There are three main reasons why exercises play pivotal role in improvement of insulin sensitivity. Firstly, during exercise, capillary perfusion increases, which leads to an elevated muscle glucose uptake. Secondly, physical activity increases the sarcolemmal and t-tubular transport of glucose from the interstitium to the muscle, which is supported by the increased content of GLUT4 in the cell membrane. Thirdly, physical exercise activate molecular mechanisms that lead to GLUT4 translocation to plasma membrane [134,135]. Studies in animal models have shown that aerobic training improves insulin sensitivity as evidenced by a decrease in value of the homeostatic model assessment of insulin resistance (HOMA-IR) or Lee index in trained animals compared to the sedentary group [136,137]. 

Similar results have been achieved in research involving human subjects. The results of these studies confirm that aerobic training increases insulin sensitivity [138,139,140]. Available data also suggest that improved glucose tolerance and whole-body insulin sensitivity may be caused even by short-term aerobic exercise training [141]. Available data indicate that physical activity has a beneficial effect on many proteins in the insulin pathway. Physical training improves insulin-stimulated glucose uptake by muscle via increases in the expression and/or activity of proteins involved in intracellular insulin signaling pathways. Exercise has been shown to increase both, the content and the phosphorylation of insulin receptor [142]. In addition, it is well known that phosphorylation of tyrosine residues in the IRS allows for proper signal transduction of insulin, whereas phosphorylation of serine residues inhibits the insulin pathway. Da Silva’s studies have found that even a single bout of physical exercise improves insulin signaling by inhibiting the phosphorylation of serine residues and increasing the phosphorylation of tyrosine residues in IRS1 [143]. Moreover, performed studies indicate that exercises also increase mRNA level of PI3-kinase [144]. PI3K through Akt/PKB, not only affects the increase of glucose uptake but also the increase in glycogen synthesis [145]. 

It has been noticed that even a single bout of exercise causes an intense increases in glucose uptake into skeletal muscle, lasting up to several hours post-exercise. The muscle contraction promotes GLUT4 translocation from intracellular sources to the sarcolemma and T tubules which increases the number of sites at which glucose can be transported into the muscle [146,147,148,149,150]. Moreover, it has been shown that exercises, in addition to increasing GLUT4 translocation to the cell membrane, also lead to an increase the intracellular level of this protein. Endurance training leads to an increase in GLUT4 expression in both human and animal skeletal muscles [144,151,152,153]. Furthermore, it has been demonstrated that aerobic training is a potent inducer for GLUT4 enhancer factor (GEF), resulting in increased GLUT4 expression and improving glycemic control [154]. It has been found, that single bout of exercise also increases AS160 level [155] and Akt/PKB threonine phosphorylation [155] and serine phosphorylation of this protein in rat skeletal muscle [143]. This leads to enhancement of insulin-stimulated glucose transport by increasing GLUT4 translocation to plasma membrane [155]. Moreover, studies conducted on healthy young men showed the beneficial effect of three-weeks’ exercise on the increased expression and activity/phosphorylation of Akt and AS160 [156]. Physical activity not only increases glucose uptake, but also glycogen synthesis by affecting the regulation of glycogen synthase (GS) activity. Studies in humans have shown that physical training significantly increases GS activity in both healthy and type 2 diabetes subjects [157]. During exercise, GS activity is affected by both stimulating and inhibitory factors, therefore, the effect of exercise on GS activity is a result of the relative strength of various stimuli [135]. In conclusion, physical training increases glucose uptake, as well as glycogen synthesis in the skeletal muscle with no change in glycogen synthase expression [142,158]. It has also been revealed that low intensity strength training leads to an increase in the content of GLUT4 protein and insulin receptors [159,160]. Improvement in insulin-stimulated glucose transport in aerobic- and resistance-trained animals results from, among other things, an increase in the basic isoform of insulin-sensitive glucose transporter (GLUT4) [124]. Yaspelkis et al. found in their studies that the level of GLUT4 protein in aerobic-trained animals was increased in lower leg muscles, which are mainly used for running (i.e., soleus muscles, plantaris muscle, red gastrocnemius muscle) [124]. In turn, in animals after resistance training, the GLUT4 content was higher in the muscles of the upper limb that are needed to perform a squat (i.e., the red-and-white quadriceps muscles). Taken together, these data confirm that the mechanisms of action of different forms of exercise in improving glucose homeostasis are similar.

### 3.2. Physical Activity and Lipid Metabolism

The effect of exercise on increase in insulin sensitivity is largely caused by an improvement in lipid metabolism. One of the main effects of physical activity is a reduction in fat mass, especially in the visceral region, associated with a significant reduction in obesity. This improves many metabolic disorders associated with obesity, including insulin resistance [161,162]. Both types of exercises, resistance and aerobic, cause reduction of subcutaneous and visceral adipose tissue [163]. The improvement in body composition resulting from physical activity also has a positive effect on obesity-related inflammation [164]. It has been found that the level of circulating inflammatory mediators released from adipose tissue, in particular TNFα-1 and IL-6 [165,166,167,168], decreases with weight-reducing exercises [121]. 

It is worth noting that weight loss in obesity also has a positive effect on hormonal balance. A study by Milan et al. [169] proved that weight loss in obese rats increased the expression of adiponectin in visceral adipose tissue. All these changes were accompanied by an improvement in insulin sensitivity and thus higher glucose uptake. It would seem that the improvement in insulin sensitivity caused by the reduction of inflammation or the restoration of hormonal balance is only the result of prolonged exercise that is associated with weight loss. However, studies have shown that the insulin sensitizing effect of exercise is maintained independently of weight loss and fat redistribution [138]. In addition, studies by Koh et al. have shown that aerobic training can reduce inflammation without decreasing total fat mass [170]. It has also been demonstrated that aerobic exercise significantly reduces TNF-α concentration while maintaining the initial body weight. Interesting results were presented by Nassis et al. [138] who showed that despite a 12-week aerobic training, girls who were overweight or obese significantly increased their insulin sensitivity. However, what is more important, these changes occurred with the maintaining the initial weight, the same percentage of fat, and the unchanged concentration of circulating adiponectin, IL-6, CRP, and other inflammation markers. The data presented here suggest that increased physical activity may ameliorate the metabolic disturbances associated with obesity.

An interesting phenomenon, associated with regular physical activity, is called athlete’s paradox. Insulin resistance and T2D is associated with increased intramuscular lipids content. A higher amount of these lipids is also observed in endurance-trained athletes, who have high oxidative capacity and who have enhanced insulin sensitivity [171]. It has been demonstrated that physical activity increases the expression of the genes responsible for triacylglycerols [172] synthesis, which in turn leads to the accumulation of TAG in the muscles. The higher level of intramuscular TAG in athletes acts as an adaptive response to endurance training. It allows for a greater contribution of the lipid pool as a substrate source during prolonged exercise [173,174]. Similar results were obtained by Dube et.al [171] who studied the effect of training on intramuscular lipid levels, muscle oxidative capacity and insulin sensitivity in previously sedentary overweight or obese, insulin resistant older subjects. Data obtained by these researchers showed that training significantly increased the level of intramuscular lipid, but at the same time caused a decrease in the content of diacylglycerols and ceramides. These results showed that physical activity in overweight older people improves insulin sensitivity with a decrease in the content of biologically active lipids [171]. It has also been proven that single bouts of exercise reduce the level of intramuscular LCACoA in animals fed a high fat diet, which was accompanied by higher insulin-stimulated muscle glucose uptake and increased whole body insulin sensitivity. 

Additionally, in an animal model, two hours of swimming has been shown to significantly reduce the level of malonyl-CoA, a key intermediate in the fatty acids synthesis, which is believed to be an important factor inducing muscle insulin resistance due to its ability to inhibit CPT activity [41,175]. CPT is responsible for the transport of long chain fatty acids across the mitochondria membrane for β-oxidation and malonyl-CoA inhibits CPT1 activity and, thus, also mitochondrial fatty acid transport [176]. In work, where the effect of the duration of a single bound of exercise on ceramide metabolism in skeletal muscle was examined, it was shown that the content of ceramide in muscles is influenced by both, the duration of exercise and the type of muscle. It has been found, that after 30 min of exercise, muscle ceramide content was significantly lower than in control, sedentary animals. 

Moreover, it has been demonstrated that expression and activity of serine palmitoyltransferase (SPT), a key enzyme of sphingolipid metabolism, as well as the content of sphinganine, a main intermediate of de novo ceramide synthesis increased gradually with the duration of exercise [177]. In human studies, conducted on three groups of people: obese volunteers, endurance trained athletes and people with type 2 diabetes, it has been shown that at rest, total muscle ceramide content did not differ between all groups, however, the content of C18:0-Cer in T2D patients was significantly higher than in athletes and it was positively associated with BMI and inversely related to insulin sensitivity [178]. Moreover, in all groups, acute exercise induced a considerable increase in transcription of genes encoding sphingolipid synthesis enzymes as well as the total ceramide content. At the same time, the largest increase after acute exercise was observed in C18:0-Cer levels and this compound was significantly higher in obese and type 2 diabetic subjects than in athletes. In addition, it has been observed that ceramide content decreased after recovery. These data imply that ceramide and other sphingolipids containing stearate are especially connected with insulin resistance in skeletal muscle [178]. Reduced ceramide synthesis during recovery after acute physical activity, reduces muscle ceramide content, which may be the reason for increased insulin sensitivity caused by a single bout of exercise [178]. Another work has shown that aerobic training also affects sphingolipid metabolism. It has been reported that, in animal muscles, training increased the activity of enzymes responsible for ceramide synthesis, but the level of ceramides has not changed [136]. However, human studies have shown that training caused a decrease in ceramide content in skeletal muscle [179]. Importantly, these changes in intramuscular lipids were correlated with the observed improvement in glucose tolerance [179]. Schenk and Horowitz have shown that a single session of exercise in humans can improve insulin sensitivity, which is likely due to the observed significant decrease in diacylglycerol content as well as tendency to reduce ceramide concentration [173]. In addition, it has been shown that the protein expression of sterol CoA desaturase 1 (SCD1), which desaturates fatty acids and enhances their incorporation into triglycerides, was significantly higher in exercisers and as a result, the level of TAG increases [173]. Other studies have shown that endurance training significantly improves glucose tolerance by changing the lipid metabolism in muscle [179]. Training has been shown to inhibit the activity of acyl-CoA carboxylase (ACC), which is responsible for the synthesis of malonyl-CoA. This effect is the result of the activation of an important enzyme that regulates lipid metabolism—AMP activated protein kinase (AMPK). This reduces the level of malonyl-CoA, a CPT inhibitor, thus increasing CPT1 activity [180]. AMPK is an enzyme involved in glucose transport, lipid and protein synthesis, and regulation of factors associated with insulin resistance (IRes) [181]. Therefore, dysregulation of AMPK can play a relevant role in the pathogenesis of IRes, metabolic syndrome and related diseases [182].

## 4. Conclusions

**Figure 2:**
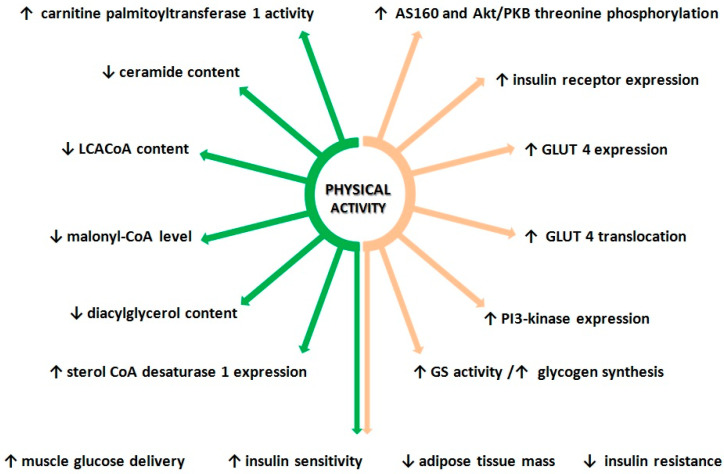
Effects of physical activity on lipid metabolism and insulin sensitivity. Orange arrows—impact of exercise on lipid metabolism; green arrows—impact of physical activity on glucose metabolism.

The data presented above demonstrates positive effect of physical activity on glucose metabolism. Exercises affect the insulin signaling pathways by increasing expression and activity of key proteins known to regulate glucose metabolism in skeletal muscle. This positive effect of exercise on muscle insulin pathway activity, at least in part, is due to changes in lipid metabolism (Figure 2). Research demonstrates positive effect of physical activity on, healthy and insulin-resistant individuals of all ages [183,184,185]. Current studies show, that the increment in insulin sensitivity after aerobic training was present in younger people [186]. Older people require more intensive/frequent or longer exercise training programs to improve insulin sensitivity [187]. The comprehensive impact of physical exercises is the reason why they are recommended for both the prevention and treatment of insulin resistance and type 2 diabetes.
